# Validation of an Informant-Reported Web-Based Data Collection to Assess Dementia Symptoms

**DOI:** 10.2196/jmir.1941

**Published:** 2012-03-12

**Authors:** Kenneth Rockwood, An Zeng, Chris Leibman, Lisa Mucha, Arnold Mitnitski

**Affiliations:** ^1^DGI Clinical IncHalifax, NSCanada; ^2^Department of MedicineDalhousie UniversityHalifax, NSCanada; ^3^Janssen Alzheimer Immunotherapy R&D, LLCSouth San Francisco, CAUnited States; ^4^Pfizer IncCollegeville, PAUnited States

**Keywords:** Dementia, online survey, symptoms, Dependence Scale, staging, cognitive impairment not dementia, mild cognitive impairment, validation, World Wide Web

## Abstract

**Background:**

The Web offers unprecedented access to the experience of people with dementia and their care partners, but data gathered online need to be validated to be useful.

**Objective:**

To test the construct validity of an informant Web-based data collection to assess dementia symptoms in relation to the 15-point Dependence Scale (DS).

**Methods:**

In an online survey posted on the DementiaGuide website, care partners of people with dementia built individualized profiles from the 60-item SymptomGuide and completed a questionnaire, which included the DS and a staging tool.

**Results:**

In the 250 profilees (155, 62% women, mean age 77 years), increasing dependence was associated with a greater chance of institutionalization. For example, no one at the lowest levels of dependence (DS score < 5, n = 33) was in long-term care, compared with half (13/25) of the profilees at the highest levels of dependence (DS score > 12) being in institutions (χ^2^_4_ = 27.9, *P* < .001). The Web-based DS was correlated with the number of symptoms: higher DS scores were associated with a higher stage of dementia (*F* > 50, *P* < .001).

**Conclusion:**

In an online survey, the Web-based DS showed good construct validity, potentially demonstrating how the Web can be used to learn more about dementia progression and how it relates to symptoms experienced by patients across the course of dementing illnesses. Even so, caution is needed to assure the validity of data collected online.

## Introduction

Dementia is a progressive disorder that affects memory, thinking, language, judgment, and behavior. This high dimensionality challenges measurement of the myriad effects of progressive neurocognitive illnesses. Our group has aimed to understand dementia by focusing on a few symptoms of greatest importance to each patient [1]. This focus on how patients experience dementia is achieved through an individualized approach, in which each patient–care partner dyad focuses on just a few symptoms (typically 3–6) of most importance to them. Individualization is feasible in clinical trials. It offers a means of evaluating medications by how well they work on dementia symptoms, and not just on how much they improve scores on standardized tests [2,3].

A more common—and complementary—way to reduce dimensionality in dementia and achieve some sort of quantitative understanding of a patient’s overall disease course is by means of staging the dementia. This can be done from semi-individualized clinical interviews, or by focusing on functional disability as a means of understanding the impact of dementia. Instruments such as the Functional Assessment Staging Tool describe impairment in self-care needs and instrumental activities; such disability is highly correlated with the other 10 axes (including memory, orientation, language, praxis, and behavior) of the Brief Cognitive Rating Scale [4]. Staging of dementia severity also typically reflects increasing dependence on others—not just for disability, but also because impaired judgment can pose risks. As a complement to classic staging instruments, the Dependence Scale (DS) was developed to measure the amount of care required by dementia patients [5]. Longitudinal studies of change in DS scores have demonstrated its validity and reliability in reflecting increased impairment with dementia progression [6].

Dependence is also well understood by those who care for people with dementia, making it a potentially useful construct in surveys. Such surveys offer some important potential in helping to bridge the gap between the lived experience of dementia and its measurement in clinical research, especially clinical trials, where the issue of clinical relevance can be fraught [2,7]. The popularity of the World Wide Web enables patients and their caregivers to conveniently access the Internet, and many do, especially adults caring for aging parents [8]. A 2011 survey found that 59% of American adults reported looking for health information online; this included 88% of self-reported caregivers. Overall, 17% of all Internet users reported looking online for information about memory loss, dementia, or Alzheimer disease [9]. Even so, how to make best use of this access remains unclear. If it unifies understanding of disease progression, the concept of progressive dependence might help translate from the clinical endpoints employed in trials to better understand the clinical meaningfulness of what historically appear to be small changes in neuropsychological test scores. To do so, it is important to be able to evaluate the information provided by respondents online. Here, our primary aim was to test the construct validity of Web-based symptom profiles. We compared these profiles with a responder-completed assessment of a patient’s level of dementia using the DS and an informant-reported staging tool. In particular, we expected to see more symptoms at higher levels of dependence, and to see more symptoms in those in institutions than otherwise. In addition, we examined patterns among the symptoms that patients targeted and their DS score, expecting to see more behavioral symptoms and functional dependence symptoms with increasing levels of the DS.

## Methods

### Setting

This is an online survey of visitors to the DementiaGuide website [10]. That site’s SymptomGuide is a Web-based tool for persons with dementia and their caregivers to identify the symptoms they are exhibiting and track how the symptoms change over time [1]. The 60 dementia symptoms in the online symptom library (Multimedia Appendix 1) and the corresponding hundreds of plain-language descriptors are easily understood by families and caregivers, who for the most part are the chief users of the site. For each symptom, information is available about its definition and descriptions, the typical stage of dementia at which it occurs, and common management strategies. These are accessed by clicking on tabs visible on each symptom, a tab providing standard advice from a physician about the typical challenges and course related to that problem, and another tab (“What’s happening in the Brain?”) that describes the pathophysiology in lay terms.

In an open survey, participants were sampled from website visitors who visited the site long enough to view its symptom library. Participants who completed the survey were offered a free subscription to the website for their participation. An announcement of the survey was posted on the DementiaGuide homepage and sent to registered site users via email. There was no other advertising for the survey.

### Measures

Visitors (caregivers) to the DementiaGuide website [10] were invited to complete a 3-part care survey. The survey was developed from existing measures, as follows. First, we asked basic information related to caregivers and the person they care for, such as age, gender, type of dementia, and living arrangements. The second part listed the 13 questions of the DS [5,6] to assess the level of care. The DS questions were exactly as they appeared in the print version of the questionnaire. The final part provided specific information about the 60 symptoms, from which people noted which symptoms were present in the person for whom they cared, and so provided an individualized symptoms profile. In addition, they staged dementia qualitatively, using the grades very mild, mild, moderate, severe, and very severe (Multimedia Appendix 2). After completing the symptom profiles, caregivers assayed the stage of the person whom they were profiling using these descriptors.

In addition to cognitive symptoms (eg, impaired recent and remote memory, expressive language, naming, understanding, attention, and orientation to time and place) SymptomGuide symptoms included disability in instrumental and personal activities of daily living (ADLs), and behavioral and psychological symptoms of dementia (BPSD). SymptomGuide tracks other common symptoms, typically related to a general construct of function, which often go unmeasured in clinical trials (eg, looking after grandchildren, operating appliances, hobbies, leisure activities, planning, and social engagement). Note that, in contrast to typical use of the SymptomGuide, where only those symptoms targeted for treatment are selected as part of a patient profile, here we asked users to note any symptoms that were problems in relation to the person for whom they cared.

The DS was described in 1994 [5]. It is based on the notion that patient dependence and the need for supervision is a means of unifying disease progression from the cognitive, functional, and behavioral standpoints [5,11,12]. It is scored by an algorithm that counts responses to 13 questions about patient dependence and need for care and supervision. The DS score ranges from 0 to 15, with higher scores indicating a higher care burden. It has been cross-validated in several clinical settings [6,13-16], including in a prospective longitudinal study [5]. The DS’s interrater reliability was high (intraclass correlation coefficient = .90) as was the internal consistency (eg, Cronbach alpha of .66–.93, depending on the subscales). Although it has not been validated in an online environment, its apparent ease of use, as well as its measurement properties, makes it an attractive way to provide a standardized description of people with dementia who might be the object of dementia surveys. All 13 items on the DS questionnaire [5,6] could be represented by similar symptoms in the SymptomGuide.

The survey was displayed over 3 screen pages. Completeness checks were done for each individual page, with unanswered questions highlighted for the participant, with completion prompted before going to the next page or submitting the survey. Respondents could hit the back browser button if they wished to edit a previous response page. The Internet protocol address of study participants was captured, and duplicates were filtered in the analysis, with only data from the first completed survey used. No cookies were used. A timestamp was captured after the first page was completed, but no time cut-off was used. Survey functionality was tested on a development site before moving to the live site.

### Analysis

Given a 95% completion rate (below), we analyzed only completed questionnaires. Validity was assessed chiefly by construct validation as follows. As the DS represents increasing dependence, we compared it against the proportion of people institutionalized by DS group; the hypothesis was that as the DS increased, so did the chance of being in assisted living or in institutional long-term care. DS scores were plotted against the severity reported on the SymptomGuide, operationalized as the number of symptoms endorsed. (Again, we expected a positive relationship.) The data distribution was first inspected for linearity; if linear, a regression analysis was performed; otherwise analysis of variance (ANOVA) was used to measure group mean differences.

To compare DS scores by user-rated grades of severity, we constructed a box-and-whiskers plot. A Spearman rank-order correlation was used to assess the values of the DS in relation to the 5 severity levels. We conducted separate ANOVAs for increasing dependence in instrumental and personal ADLs and in BPSD.

To analyze the relationships between the DS score and dementia stages identified by the participants as very mild, mild, moderate, severe, and very severe (based on symptom profiles and function, Multimedia Appendix 2), we calculated the distributions of people by the DS for each severity group. The numbers of profilees at a given DS score were cross-tabulated with the stage of dementia that was assigned by each profilee’s caregiver. These distributions were smoothed using a moving average and indicate the probabilities for an individual with a given DS score to belong to any given severity group. We calculated empirically derived crossovers of overlapping distributions from the ratios of probabilities (likelihoods) to define cut points for each DS severity interval. ANOVA was conducted to assess associations between the DS scores and the stages of dementia.

### Ethics

All respondents to the survey consented by checking their agreement to the terms and conditions of DementiaGuide, which includes their consent to the use of anonymized data. No personal information was collected that could identify the survey participant. All responses are stored on a secure server.

## Results

From January 27, 2010 to August 24, 2010, of 514 unique visitors who viewed the symptom library, 264 started the questionnaire and 250 respondents completed the online survey (completion rate 94.7%; net response rate 48.6%). All were care partners, completing information about symptoms in patients (profilees). Of the 250 care partner respondents, most were women (203, 81.2%), 208 (83.2%) and were less than 65 years old, usually with adult children (125, 50.0%) or spouses (71, 28%). Most care partners lived in the same household (115, 46.0%) or saw the person with dementia at least 5 days a week (46, 18%) although 18 (7%) saw the person less than weekly. Most care partners came from the United States (113, 45.2%), Canada (82, 33%), or the United Kingdom (22, 9%). The mean age of the patients being profiled was 77.1 years (SD 11.1) and 155 (62%) were women. Most (198, 79.2%) were community dwelling, with the remainder (52, 21%) in assisted living or nursing home care. DS scores ranged from 0 to 14, with a mode of 8.

With respect to patient residence (community versus institution), none of the 33 people profiled at the lowest levels of dependence (DS score < 5) lived in a long-term care facility, whereas of the 25 profilees at the highest level of dependence (DS score > 12) 13 (52%) were resident in a long-term care facility (χ^2^_4_ = 27.9, *P* < .001).

**Figure 1:**
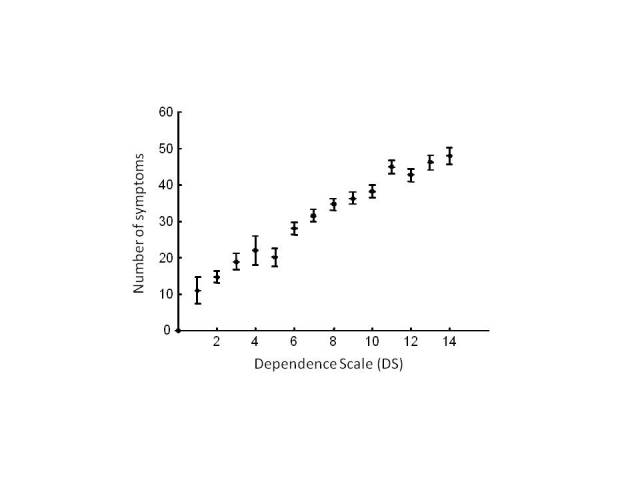
Mean number of dementia symptoms as a function of the Dependence Scale score. Circles show the means and bars show the standard errors.

**Figure 2:**
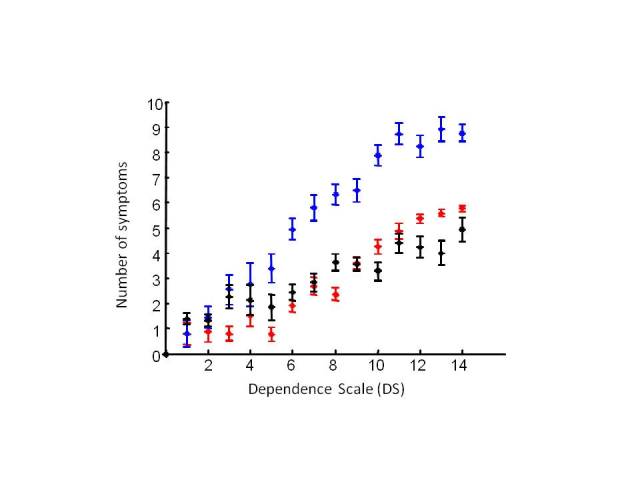
Mean number of dementia symptoms: instrumental activities of daily living (blue), activities of daily living (red), and behavioral and psychological symptoms (black). Given that only 5 people had a Dependance Scale score of 0 or 1, these states are combined here.

Profilees who experienced more symptoms tended to be more dependent in general (Figure 1). The number of the SymptomGuide symptoms and the DS score were highly correlated (*r* = .73, *P* < .001). Likewise, as the DS score increased, so did the number of instrumental and personal ADL symptoms and the number of BPSD (*F*_4_ = 76, *F*_4_ = 54, and *F*_4_ = 14, *P* < .001; Figure 2). Note that the personal ADL and BPSD types of symptoms were especially uncommon at DS scores <5. The mean number of targeted instrumental ADL symptoms increased from 1 at DS scores < 2 to 8 at DS scores > 10.

**Figure 3:**
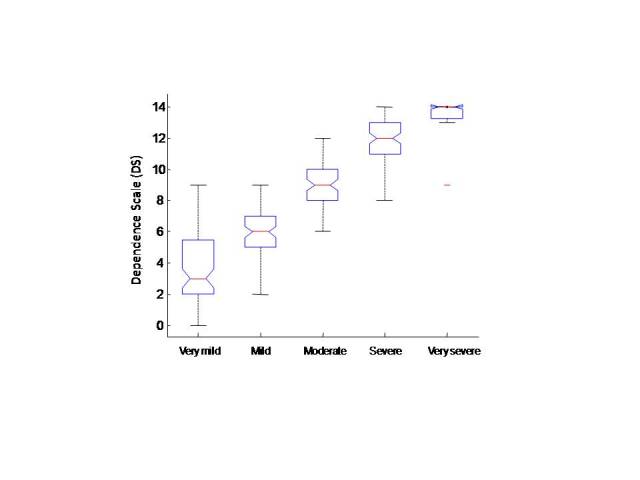
Dependence Scale score by grade of severity as a box-and-whiskers plot. The boxes show medians (red lines), edges at the quartiles Q1 and Q3; the whiskers show the boundary for the outliers.

The DS score also increased as the user-based severity staging score increased (*r* = .85, *P* < .001; Figure 3). ANOVA confirmed a significant difference in the association of the DS score by severity groups (*F*_4_ = 148, *P* < .001; Figure 3).

**Figure 4:**
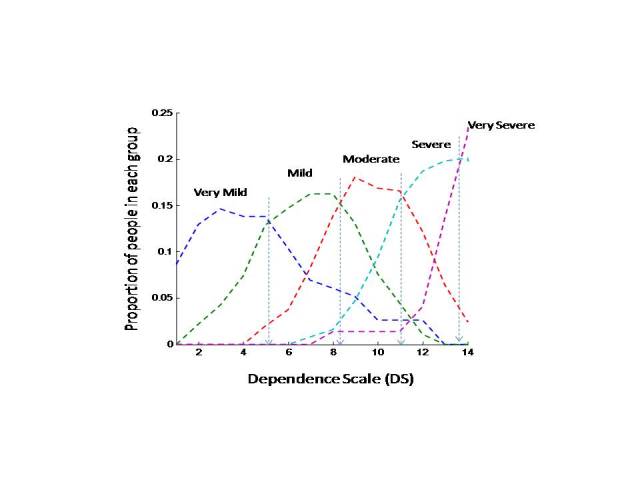
Probability distribution of the Dependence Scale score by dementia stage. Arrows indicate crossovers between the neighboring distributions and thus represent the the cut points to distinguish between the stages.

We discerned 5 empirically derived cut points at ≤5, ≤8, ≤11, ≤13, and >13; the modes increased with increasing values of the DS (Figure 4) as did the median values (ie, ≤5, median proportion value of the DS; ≤8; ≤11; ≤13; >13). We chose the cut points by using the crossovers between the neighboring distributions as indicated in the legend of Figure 4. The distributions are not normal (at least on the extremes, the medians are deviate from the means); the crossover’s location, however, indicates that on the right from the crossover, the probability of the right distribution is higher than that on the left. It could be said that the cut points were chosen in accordance with the maximum likelihood of belonging to one or the other group. Note that when the DS score was <2, profilees were almost all in the stage of very mild dementia. When the DS score was between 2 and 8, most profilees were in the mild dementia stage. Profilees identified as having moderate dementia had a DS score varying from 6 to 12. When the DS score was >10, the profilees were predominantly at the severe or very severe stage of dementia, and no one remained in the mild stage.

## Discussion

This study used an online survey of dementia caregivers and compared how they scored the DS with how they completed a checklist of 60 dementia symptoms and how they staged dementia severity. Most respondents came from the United States, Canada, and the United Kingdom and were women profiling people with dementia, chiefly parents and spouses. Several analyses (correlation of the DS score with the total number of symptoms and those specific to functional dependency and BPSD; comparison of proportions of patients receiving institutional care by DS grade; and comparison with a staging instrument) suggest construct validity in online use of the DS.

Our data must be interpreted with caution. Consistency of like measures is an important aspect of construct validity. Even so, it does not guarantee that the profiles portrayed here are what an independent observer would find. This potential weakness is shared with other self-report data and not just those collected online. On the other hand, to the extent that care partner reports tap an aspect of dementia profiles that are comparatively understudied, this may be a necessary trade-off to gather more data on the views of caregivers and what is important to them. For example, many people who use the website set goals in relation to repetitive questioning, as they did in clinical trials in which this option was available to them [17-19]. Even so, this problem receives almost no attention in the current clinical trial environment. What is more, informant-based reports have a long history in understanding dementia staging in epidemiological surveys, leading to a call for their use in electronic formats [20]. Building on this experience with self-report in standardized instruments, the World Wide Web offers a means for care partners to draw attention to what is important to them, as a way of sensitizing researchers about the lived experience of dementia. At the same time, it must be remembered that the Web tends to be used less by older people, people with lower socioeconomic status, and those with lower levels of education, who are less likely to participate in Web-based medical programs [21]. In addition, a comparatively high proportion (21%) of the people described here were in assisted living or nursing homes, with correspondingly higher levels of dependence. Given such considerations, we underscore that this cannot be seen as a representative survey, which is why we have been careful not to make any such claim. Still, it is interesting to note that our respondents, in being women, usually spouses or adult daughters, living with or near the person with dementia, are not in these ways dissimilar to typical dementia caregivers in Western countries [22].

In addition, this approach is relatively new, so it is not clear where the magnitude of the correlations—which are moderate to high by traditional standards—fits in the online environment. We also cannot be sure that the respondents were describing real cases, although this would be true also for postal or telephone surveys, so that the same cautions as exist with these better-accepted forms of data collection would need to apply here. For example, we cannot make prevalence estimates, although it remains possible to study associations between variables.

Our data potentially contribute to understanding how the Web might be used to gather information about dementia. At present, the Internet is being used in several contexts in dementia. For example, it is used to elicit opinions about issues related to Alzheimer disease (such as the merit in screening for it) [23] and to collect data about the burden of care [24,25]. The Internet is also being used to deliver care interventions [26-28] and to provide educational programs to formal care providers [29]. Note too that, while several of the interventions or surveys are targeted to particular groups, more widespread uses include the identification of people who might be at an increased risk of dementia [30]. Recently, too, the Web has been used as a means of doing experiments, such as testing whether people who took up cognitive training in the form of video games showed improved cognitive functioning on tests related to the items practiced in the games [31]. Such potential obliges designers to maximize the usability of their sites, and in particular to clearly identify the source of their information [32].

The information here is also more than just correlation. For example, the mismatch between DS scores and caregiver impressions of staging in relation to a DS score of 9,where 70% (23/33) were given a moderate stage by the caregiver, 6% (2/33) severe, 3% (1/33) very severe, 9% (3/33)very mild, and 12% (4/33) mild, illustrates a potential problem with the DS. In getting data on the views, if not of patients themselves, then of people involved with them on a frequent basis, who were asked to consider which symptoms they displayed, we are helping to meet a gap in understanding. From a time when people with dementia were not considered able to contribute to descriptions of the syndrome because of the nature of their illness, it became clear that their perspective was particularly important to understanding how people with dementia cope [33].

That this perspective is important is clear if we consider that, although the literature on the lived experience of dementia is rich [34,35] it has had comparatively little direct impact in the clinical trials literature on the meaningfulness of dementia treatment [36]. The perspective of individuals on how they cope has a clear impact on the response to psychosocial therapy [37]. This has led to calls for a better understanding of how both pharmacological and nonpharmacological treatments might have an impact in ways that are evident to patients and their care partners [38,39]. This will be especially important in the longer trials needed to evaluate the impact of potentially disease-modifying therapies, as these cover a span in which patients’ perceptions of quality of life can change [1,40]. How symptoms change over time from the perspective of care partners is an important concern that is motivating additional inquires by our group.

## Multimedia Appendix 1

List of 60 symptoms.

## Multimedia Appendix 2

Operationalizing dementia staging in the online questionnaire.

## Abbreviations

ADL: activities of daily living
ANOVA: analysis of variance
BPSD: behavioral and psychological symptoms of dementia
DS: Dependence Scale
