# Supplementary material for: Validation of an Informant-Reported Web-Based Data Collection to Assess Dementia Symptoms
Source: J Med Internet Res. 2012 Mar 12;14(2):e42. doi: 10.2196/jmir.1941 (PMC3376520; doi:10.2196/jmir.1941)
Supplement: Supplementary file 1 [file jmir_v14i2e42_app1.pdf]

Multimedia Appendix 1. List of 60 symptoms

	Symptom's name
1	Incontinence
2	Mobility
3	Impaired Sensory Input
4	Balance Problems
5	Problems with Eating
6	Physical Complaints
7	Impaired Attention/Concentration
8	Impaired Comprehension/Understanding
9	Problems with Decision Making
10	Inappropriate Language and Behaviour
11	Impaired Judgment
12	Unsafe Actions
13	Problems Following Instructions
14	Insensitivity
15	Repetitive Questions/Stories
16	Language Difficulty
17	Impaired Memory for Names and Faces
18	Past Memory Impairment
19	Memory of Recent Events
20	Misplacing or Losing Objects
21	Reading or Writing
22	Needs Help with Bathing
23	Needs Help with Dressing
24	Problems with Driving
25	Problems with Telephone Use

26	Problems with Financial Management
27	Problems with Household Chores
28	Difficulty with Meal Preparation/Cooking
29	Problems Operating Gadgets/Appliances
30	Poor Personal Care/Hygiene
31	Needs Help with Shopping
32	Sleep Disturbances
33	Aggression
34	Delusions and Paranoia
35	Disorientation to Place
36	Disorientation to Time
37	Hallucinations
38	Inappropriate Sexual Behaviour
39	Irritability/Frustration
40	Repetitive Behaviour
41	Decreased Appetite
42	Low Mood
43	Obsessive Behaviour
44	Wandering
45	Anxiety and Worry
46	Problems with Insight (Others/Environment)
47	Decreased Interest/Initiative
48	Low Self Esteem
49	Restlessness
50	Impaired Independence
51	Personality Changes
52	Impaired Self-Awareness

53	Activities with Other People
54	Decline in Hobbies
55	Problems with Interaction with Friends and Family
56	Interaction with Strangers
57	Needs Help with Looking after Grandchildren
58	Social Interaction/Withdrawal
59	Problems with Spirituality and Religion
60	Problems in Travel and Vacationing
