# Supplementary material for: Validation of an Informant-Reported Web-Based Data Collection to Assess Dementia Symptoms
Source: J Med Internet Res. 2012 Mar 12;14(2):e42. doi: 10.2196/jmir.1941 (PMC3376520; doi:10.2196/jmir.1941)
Supplement: Supplementary file 2 [file jmir_v14i2e42_app2.pdf]

Multimedia Appendix 2. Operationalizing dementia staging in the online questionnaire.

Of the following 5 stages of dementia, at which one would you place the person you care for?

- Very mild - e.g. has trouble with work or complicated hobbies (such as wood working, cross stitching, cross word puzzles)
- Mild - e.g. has problems with banking, making meals, playing card games, house work, using the computer
- Moderate - e.g. needs prompting in choosing appropriate clothing to wear, or needing prompting or supervision with personal hygiene
- Severe - e.g. needs help dressing and grooming, walking, going to the bathroom
- Very severe - e.g. able to speak only a few words; mobility is limited, is mostly in bed or a chair
